# Real-Time Fluorescence Detection of ERAD Substrate Retrotranslocation in a Mammalian In Vitro System

**DOI:** 10.1016/j.cell.2007.03.046

**Published:** 2007-06-01

**Authors:** Judit Wahlman, George N. DeMartino, William R. Skach, Neil J. Bulleid, Jeffrey L. Brodsky, Arthur E. Johnson

**Affiliations:** 1 Department of Biochemistry and Biophysics, Texas A&M University, College Station, TX 77843, USA; 2 Department of Chemistry, Texas A&M University, College Station, TX 77843, USA; 3 Department of Physiology, University of Texas Southwestern Medical Center, Dallas, TX 75390, USA; 4 Department of Biochemistry and Molecular Biology, Oregon Health & Science University, Portland, OR 97239, USA; 5 Faculty of Life Sciences, University of Manchester, Manchester M13 9PT, UK; 6 Department of Biological Sciences, University of Pittsburgh, Pittsburgh, PA 15260, USA; 7 Department of Molecular and Cellular Medicine, Texas A&M University System Health Science Center, College Station, TX 77843-1114, USA

**Keywords:** CELLBIO, PROTEINS

## Abstract

Secretory proteins unable to assemble into their native states in the endoplasmic reticulum (ER) are transported back or “retrotranslocated” into the cytosol for ER-associated degradation (ERAD). To examine the roles of different components in ERAD, one fluorescence-labeled ERAD substrate was encapsulated with selected lumenal factors inside mammalian microsomes. After mixing microsomes with fluorescence-quenching agents and selected cytosolic proteins, the rate of substrate efflux was monitored continuously in real time by the decrease in fluorescence intensity as cytosolic quenchers contacted dye-labeled substrates. The retrotranslocation kinetics of nonglycosylated pro-α factor were not significantly altered by replacing all lumenal proteins with only protein disulfide isomerase or all cytosolic proteins with only PA700, the 19S regulatory particle of the 26S proteasome. Retrotranslocation was blocked by antibodies against a putative retrotranslocation channel protein, derlin-1, but not Sec61α. In addition, pro-α factor photocrosslinked derlin-1, but not Sec61α. Thus, derlin-1 appears to be involved in pro-α factor retrotranslocation.

## Introduction

In eukaryotic cells, most secreted proteins are translocated cotranslationally into the lumen of the endoplasmic reticulum (ER), where they are glycosylated, fold, form disulfide bonds, and assemble into native structures before being transported to various destinations (Johnson and van Waes, 1999; Fewell et al., 2001; Ellgaard and Helenius, 2003; Trombetta and Parodi, 2003). When a polypeptide is unable to reach a native conformation, it is usually directed into the ER-associated degradation (ERAD) pathway (Tsai et al., 2002; McCracken and Brodsky, 2003; Kostova and Wolf, 2003; Römisch, 2005; Meusser et al., 2005). Since most proteases that degrade misfolded or misassembled proteins are located in the cytosol, ERAD substrates must be transported back through the ER membrane into the cytosol for degradation. This process—the movement of polypeptides from the ER lumen to the cytosol—has been termed retrotranslocation or dislocation.

Retrotranslocation is an intrinsically complex process: the ERAD quality-control machinery must distinguish misfolded proteins in the ER lumen from proteins that are on the way to folding properly, and ERAD substrates must be targeted to a retrotranslocation site in the ER membrane, transported through the membrane, and directed to sites of proteolysis (Johnson and Haigh, 2000). Proteins in and on both sides of the ER membrane are therefore required to function together to accomplish this mechanistically difficult task. Since ERAD was first identified (Sommer and Jentsch, 1993; Ward et al., 1995; Jensen et al., 1995; McCracken and Brodsky, 1996; Hiller et al., 1996; Hampton et al., 1996), a number of proteins have been shown to be involved directly or indirectly in retrotranslocation. While the specific roles of some proteins have been determined, the functions of others are either unknown or controversial.

The confusing and sometimes contradictory data about the involvement of specific proteins in retrotranslocation result in part from limitations in the experimental approaches currently available to examine this process, especially in mammalian systems. First, in most in vivo and in vitro studies, ERAD substrates are synthesized in the cytoplasm and cotranslationally translocated into the ER lumen concurrent with ongoing retrotranslocation and degradation. Thus, approaches to study retrotranslocation are complicated by the inability to discriminate between substrates on their way into the ER lumen and those on their way out. Even the presence of a lumen- or cytosol-specific covalent modification (e.g., signal sequence cleavage, glycosylation, or ubiquitinylation) cannot distinguish between proteins that have been completely translocated and those that were trapped at some stage prior to complete translocation. These realities interfere with accurate quantification of the extent of retrotranslocation. Second, the kinetics of retrotranslocation are difficult to quantify accurately, both for the above reasons and because retrotranslocation cannot be synchronized in most experiments. Since translocation and retrotranslocation proceed simultaneously both in vivo and in vitro at unknown and perhaps variable rates, there is currently no way to prepare samples in which all substrates are in the same state at a specific time. Hence, synchronous initiation of retrotranslocation for all substrates in a sample has been impossible. Third, the role of a particular protein in retrotranslocation is difficult to ascertain in many approaches. For example, the knockout of a particular gene may alter in vivo retrotranslocation activity, but it is difficult to know whether the observed change in activity is a primary or secondary effect. Interpretations are further complicated if the loss of a specific protein is obscured by the presence of another protein that is partially or wholly redundant.

To overcome these limitations, we developed a mammalian in vitro system that allows monitoring of retrotranslocation synchronously and in real time in the absence of concurrent translocation. A purified full-length and fluorescence-labeled ERAD substrate is encapsulated into mammalian ER microsomes with a selection of purified lumenal proteins (none, one, two, or all). Upon mixing the resulting vesicles with the desired purified cytosolic proteins, a well-defined and homogeneous sample is created. The movement of substrates from the lumen to the cytosol is then detected spectroscopically to provide a continuous real-time measurement of the retrotranslocation rate. Using this biophysical approach, we report here on the individual molecular species in the cytosol, ER membrane, and lumen that are required for the retrotranslocation of one ERAD substrate from the mammalian ER.

## Results

### Experimental Design

Fluorescence-labeled ERAD substrate molecules and a known selection of lumenal components are encapsulated inside ER microsomes. When such microsomes are added to a solution containing a known selection of cytosolic components, the resultant sample is well defined biochemically, with the desired molecular species on each side of the ER membrane. The retrotranslocation of fluorescence-labeled substrates from the lumenal to the cytosolic side of the membrane is detected because the cytosol also contains fluorescence-quenching agents. The rate of substrate retrotranslocation is therefore monitored directly in real time by the reduction in sample emission intensity as the dyes covalently attached to the substrate move through the ER membrane and are exposed to the quenchers. Since all substrate molecules are initially encapsulated inside microsomes and retrotranslocation is initiated by increasing the temperature, substrate retrotranslocation is synchronized and reproducible. Moreover, by varying the lumenal and cytosolic components in parallel samples, the requirement for individual factors during retrotranslocation may be revealed by even subtle changes in the kinetics of substrate retrotranslocation.

The ERAD substrate used in this study was Δgpαf, a nonglycosylated derivative of the yeast pro-α factor mating pheromone. Because both yeast (Caplan et al., 1991; McCracken and Brodsky, 1996) and mammals (Su et al., 1993) recognize and degrade Δgpαf, it is a legitimate model ERAD substrate. Furthermore, Δgpαf is soluble; is not ubiquitinylated; and lacks disulfide bonds, carbohydrates, and transmembrane sequences. Since these structural features may complicate retrotranslocation and require additional or different mechanisms and/or components, in this initial study we chose Δgpαf to establish the minimal requirements for retrotranslocation.

### Retrotranslocation of Fluorescence-Labeled Substrate

**Figure 1:**
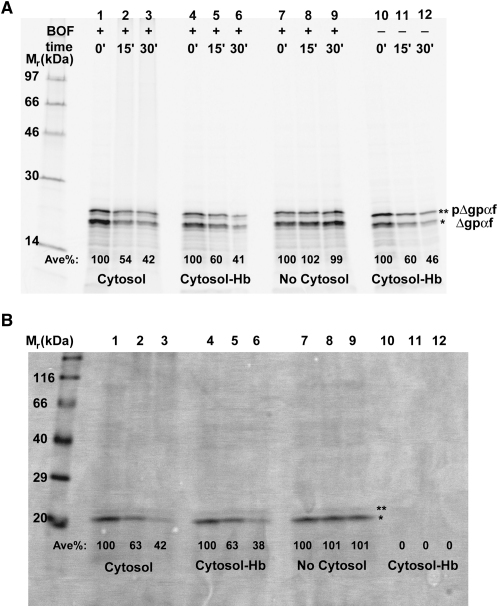
Retrotranslocation and Degradation of Δgpαf-BOF After pΔgpαf mRNA was translated in vitro in the presence of [^35^S]Met, SRP, microsomes, and either ɛBOF-Lys-tRNA^Lys^ (lanes 1–9) or Lys-tRNA^Lys^ (lanes 10–12), the microsomes were pelleted and then added to solutions containing ATP and either reticulocyte lysate (cytosol, lanes 1–3), lysate lacking hemoglobin (cytosol-Hb, lanes 4–6 and 10–12), or no lysate (no cytosol, lanes 7–9). The average percentages of Δgpαf-BOF radioactivity (A) and BOF emission (B) remaining at 15 and 30 min (0 min = 100%) for each sample are shown. (n = 7; ±3%–7% for ^35^S, ±2%–6% for BOF.) ^∗^, Δgpαf-BOF; ^∗∗^, pΔgpαf-BOF.

To determine whether the covalent attachment of a fluorescent dye to Δgpαf would interfere with retrotranslocation, we incorporated BODIPY FL (BOF) dyes into the prepro substrate, pΔgpαf, by adding N^ɛ^-(4,4-difluoro-5,7-dimethyl-4-bora-3a,4a-diaza-*s*-indacene-3-propionyl)-Lys-tRNA^Lys^ (ɛBOF-Lys-tRNA^Lys^; Woolhead et al., 2004) to an in vitro translation system. Most, but not all, of the pΔgpαf translocated into the lumen was converted to Δgpαf by signal peptidase. The efficiency of conversion was comparable to that observed in reactions employing pΔgpαf and yeast microsomes (McCracken and Brodsky, 1996; Pilon et al., 1997). After purification by sedimentation, the microsomes were added to rabbit reticulocyte lysate that contained ATP and lacked mRNA, whereupon Δgpαf exit from the microsomes was monitored (Figure 1A). The amount of radiolabeled (lanes 1–12) and BOF-labeled (lanes 1–9) Δgpαf in microsomes decreased markedly over time in samples with either complete lysate (lanes 1–3) or lysate lacking hemoglobin (lanes 4–6 and 10–12), but not in samples lacking cytosol (lanes 7–9). Cytosolic proteins were therefore required for retrotranslocation. The time-dependent decrease in total Δgpαf showed that the retrotranslocated Δgpαf was degraded, primarily by the proteasome because degradation was inhibited by lactacystin (see Figure S1A in the Supplemental Data available with this article online). The rates of degradation were similar for unlabeled Δgpαf and BOF-labeled Δgpαf whether lysates lacked or contained hemoglobin (Figure 1A). Importantly, the same conclusions were reached by monitoring BOF fluorescence rather than [^35^S]Met-labeled protein: the amount of microsome-encapsulated and BOF-labeled Δgpαf was reduced over time by incubating the microsomes in lysate with or without hemoglobin (Figure 1B, lanes 1–6), but little retrotranslocation was detected in the absence of cytosol (Figure 1B, lanes 7–9). Therefore, the covalent attachment of BOF to Δgpαf interfered with neither retrotranslocation nor degradation.

### Δgpαf-BOF and Microsomes with Defined Lumenal Contents

One advantage of the spectroscopic approach is that substrate from a single preparation of Δgpαf-BOF can be encapsulated and compared with parallel samples that have different combinations of lumenal and/or cytosolic components. This approach also eliminates substrate heterogeneity. We overexpressed and purified a recombinant Δgpαf derivative with a C-terminal hexameric histidine tag and four single-site mutations (N23Q, N57Q, and N67Q to prevent glycosylation, and Y165C for dye attachment). Since no targeting to the translocon was required, no signal sequence was present. This Δgpαf derivative was then covalently modified with BOF and gel filtered to remove unreacted dye, thereby yielding Δgpαf-BOF.

The lumenal contents of ER microsomes were extracted by exposure to pH 9.5–10.0 and separated from membranes by sedimentation (Bulleid and Freedman, 1988; Nicchitta and Blobel, 1993; Hamman et al., 1998; Haigh and Johnson, 2002; Alder et al., 2005). After the membranes were resuspended in a solution containing the components to be encapsulated (e.g., Δgpαf-BOF, ATP, and BiP), the solvent pH was reduced to 7.5 to reseal the microsomes in their original orientation (lumen inside) without any detectable loss of ribosome-binding activity. Following gel filtration to separate the microsomes from unencapsulated material, the reconstituted rough microsomes (RRMs) had a defined lumenal content.

To assess whether Δgpαf-BOF adsorbs to the outer surface of RRMs, microsomes were subjected to mock encapsulation procedures in the absence of Δgpαf-BOF and then incubated with Δgpαf-BOF to allow adsorption. After gel filtration, the BOF intensity of mock RRMs was ∼10**%** of that seen with RRMs that contained Δgpαf-BOF. Since any cytosol-exposed Δgpαf-BOF was quenched before retrotranslocation was initiated (see below), these molecules did not contribute to the spectral changes shown below.

### Fluorescence-Detected Retrotranslocation

In initial retrotranslocation experiments, microsomes contained Δgpαf-BOF, ATP, and a full complement of lumenal proteins that had been extracted, concentrated, and then reconstituted back into RRMs (complete RRMs, or “cptRRMs”). To obtain a full complement of cytosolic proteins, rabbit reticulocyte lysate was first chromatographed to remove hemoglobin because its presence interfered with fluorescence measurements. The resulting hemoglobin-free lysate, here termed “cyto,” was diluted ∼4-fold from the original lysate but still supported retrotranslocation (Figure 1A; Figure S1A) and was used in all fluorescence experiments. A solution containing cyto and ATP was designated complete cytosol, or “cptcyto.” The concentrations of ATP, cyto, and lumenal proteins required for maximal retrotranslocation were determined and used throughout this study. To detect substrate retrotranslocation, BOF-specific antibodies (αBOF) were added to the cytosol. Since no retrotranslocation occurs at 0°C (Figure S3), RRMs were mixed with the cytosolic components and αBOF on ice before retrotranslocation was initiated by raising the temperature to 30°C. Besides allowing complete mixing, the 0°C incubation allowed αBOF to quench any residual nonencapsulated Δgpαf-BOF before the reaction was initiated (t_0_).

**Figure 2:**
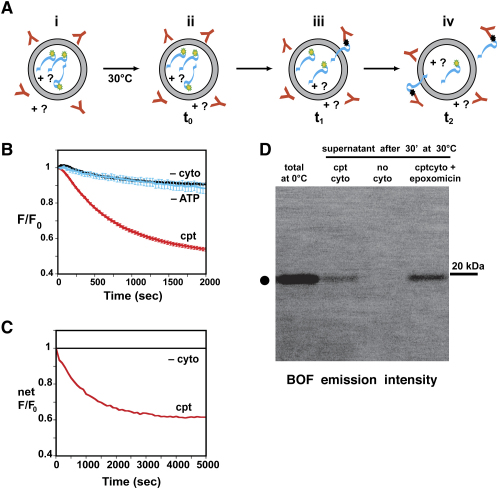
Fluorescence-Detected Retrotranslocation (A) Reconstituted rough microsomes (RRMs; gray) containing Δgpαf-BOF (cyan with yellow dye), ATP, and a selection of lumenal proteins (? = all, some, or none) are mixed on ice with ATP, αBOF (anti-BOF antibodies; red), and some selection of cytosolic proteins (? = all, some, or none) (i). Retrotranslocation is initiated at t_0_ by incubating samples at 30°C (ii). Retrotranslocation of Δgpαf-BOF from the inside to the outside of the microsomes allows αBOF to bind the BOF dye and quench its emission intensity (iii). A direct real-time measure of the rate and extent of Δgpαf-BOF retrotranslocation is therefore given by the time-dependent decrease in BOF intensity as more Δgpαf-BOF molecules are exposed to αBOF (iv). (B) The net emission intensities of samples with and without αBOF were recorded as a function of time and then normalized (F = F_+αBOF_/F_−αBOF_). F_0_ is the initial net normalized intensity at t_0_, while F is the net normalized intensity at any time t. The average F at each time point for n separate experiments is plotted; error bars in this and all other figures show the standard error of the mean for each point and dictate line thickness. αBOF-dependent quenching is shown for cptcyto (red, n = 48), ATP in the absence of cytosolic proteins (black, n = 21), and cyto lacking ATP (cyan, n = 7; 30 units/ml apyrase was included in both the lumen and the cytosol). (C) CptRRMs were incubated for an extended time with or without cytosol, and the net αBOF-dependent quenching (red, n = 3) was obtained by subtracting the −cyto αBOF-dependent quenching from the cptcyto αBOF-dependent quenching (Figure S2B). The −cyto quenching was also subtracted from itself to yield the expected flat line (black). (D) CptRRMs were incubated (30°C, 30 min) with cptcyto (lane 2), no cyto (lane 3), or cptcyto inhibited with epoxomicin (lane 4); an equivalent amount of RRMs was loaded directly into lane 1. After microsomes and encapsulated Δgpαf-BOF were removed by sedimentation, the amount of full-length Δgpαf-BOF (•) in each supernatant was determined by SDS-PAGE and imager-detected BOF fluorescence.

Since αBOF binding to BOF strongly quenches its emission, any retrotranslocation of lumenal Δgpαf-BOF into the cytosol should result in a significant reduction in sample emission intensity (Figure 2A). The magnitude of the spectral change depends on the initial state of BOF in the sample because BOF emission is partially quenched in the full-length polypeptide, and proteolysis of Δgpαf-BOF causes a 65% increase in BOF intensity (Supplemental Data). Therefore, to focus solely on αBOF-dependent intensity changes, parallel samples with or without αBOF were examined in each experiment. The magnitude of the αBOF-dependent intensity change was then given by the ratio of intensities with and without αBOF (F = F_+αBOF_/F_−αBOF_). Only the αBOF-dependent intensity is shown here.

When cptRRMs were added to cptcyto, a substantial αBOF-dependent decrease in BOF intensity was observed (Figure 2B). Maximal αBOF-dependent quenching averaged 46% ± 1% of the initial intensity after 2000 s.

### Cytosol- and ATP-Dependent Substrate Retrotranslocation

When cptRRMs were incubated in the absence of cytosolic proteins, the initial sample intensity was reduced by 9% ± 1% after 2000 s (Figure 2B). Also, when apyrase, an enzyme that hydrolyzes ATP, was included in both the lumenal and cytosolic solutions, the rate and extent of Δgpαf-BOF quenching were reduced by the same extent (10% ± 3%; Figure 2B). Thus, both cytosolic proteins and ATP are required for Δgpαf-BOF retrotranslocation, as observed in yeast systems (McCracken and Brodsky, 1996; Pilon et al., 1997; Lee et al., 2004). However, the fluorescence approach utilized here measures the kinetics of ERAD substrate exposure to the cytosol continuously at a time resolution higher than that achievable by other methods.

The small, steady decrease in Δgpαf-BOF intensity observed in the absence of either cytosol or ATP (Figure 2B) was nearly identical to the decrease observed with RRMs containing BODIPY-labeled glutathione, heavy-chain binding protein (BiP), or protein disulfide isomerase (PDI) (Figure S2A). Since BiP and PDI are ER-resident proteins and are not retrotranslocation substrates, this αBOF-dependent quenching appears to be unrelated to retrotranslocation. Subtracting this background from the observed signal change (Supplemental Data) yielded the net retrotranslocation-dependent fluorescence change (Figure 2C). Δgpαf-BOF retrotranslocation was complete when the spectral change reached a plateau, which occurred after 2000 s under our conditions.

Direct biochemical evidence for lumenal Δgpαf-BOF transport into the cytosol was provided by incubating cptRRMs with ATP and epoxomicin-treated cyto in the absence of αBOF, removing the microsomes by sedimentation, and quantifying the amount of Δgpαf-BOF fluorescence in the supernatant. On average (n = 3), 30% of full-length Δgpαf-BOF was found in the supernatant under these conditions (Figure 2D, lane 4). Much less Δgpαf-BOF was found in the supernatant when proteasomes were active (Figure 2D, lane 2), and none was found when no retrotranslocation occurred (Figure 2D, lane 3).

### PA700, the 19S Regulatory Particle of the 26S Proteasome, Stimulates Δgpαf Retrotranslocation

**Figure 3:**
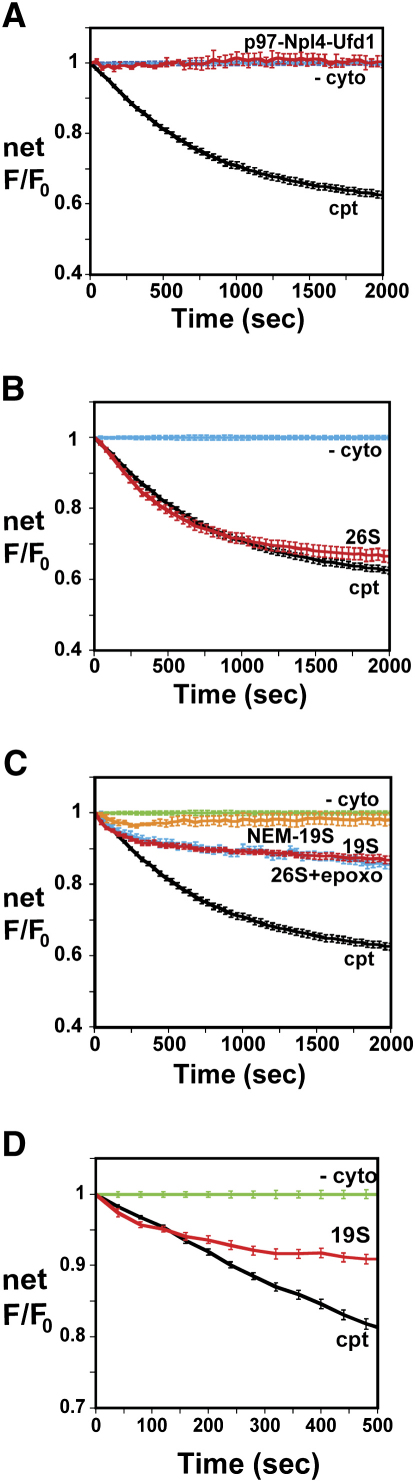
Retrotranslocation Dependence on Cytosolic Components (A–C) CptRRMs were incubated with ATP and the following: p97, Ufd1, and Npl4 (red, n = 4), cyto (black), or no cyto (cyan) (A); 26S proteasomes (red, n = 17), cyto (black), or no cyto (cyan) (B); and 19S RPs (red, n = 10), NEM-treated 19S RPs (orange, n = 4), epoxomicin-treated 26S proteasomes (cyan, n = 4), no cyto (green), or cyto (black) (C). Net αBOF-dependent spectral changes are shown after subtraction of the –cyto background (cf. Figure 2C). (D) Data in (C) shown on an expanded scale.

The ATPase complex containing p97 (VCP; Cdc48 in yeast), Ufd1, and Npl4 plays a critical role in the retrotranslocation of several ERAD substrates (Bays and Hampton, 2002; Tsai et al., 2002; Carlson et al., 2006). To assess the involvement of this complex in Δgpαf retrotranslocation, cptRRMs were incubated with purified p97, Ufd1, Npl4, and ATP. No increase in αBOF-dependent quenching over background was observed (Figure 3A). The quenching rate also did not change when excess p97 was added to cptcyto (data not shown). Thus, Δgpαf retrotranslocation is independent of p97 and its cofactors in this system.

In yeast, 26S proteasomes stimulate Δgpαf retrotranslocation (Lee et al., 2004) and possibly that of other substrates (e.g., Mayer et al., 1998; Chillarón and Haas, 2000; Walter et al., 2001). When cptRRMs were incubated with ATP and purified bovine 26S proteasomes, the maximal rates and extents of αBOF-dependent quenching were nearly the same for pure 26S proteasomes and for total cytosolic proteins (Figure 3B).

To determine whether PA700 alone can promote retrotranslocation, cptRRMs were incubated with ATP and purified bovine PA700. Since an increase in αBOF-dependent quenching was observed (Figure 3C), PA700 stimulated ATP-dependent Δgpαf retrotranslocation. Moreover, the ATPase activity of PA700 was required because pretreating PA700 with NEM (DeMartino et al., 1994) essentially blocked retrotranslocation (Figure 3C). Continuous monitoring of Δgpαf-BOF intensity revealed that the initial quenching rates were similar for samples containing either purified 26S or purified 19S regulatory particle (RP) (Figure 3D), but the total 19S-dependent increase in quenching was much less than the total 26S-dependent increase (Figure 3C).

The cause of the differential quenching between 26S- and 19S-containing samples was revealed by incubating cptRRMs with ATP and 26S that had been pretreated with the proteasome inhibitors epoxomicin or lactacystin (Gaczynska and Osmulski, 2005). The rates and extents of αBOF-dependent quenching were nearly the same for samples containing PA700 (Figure 3C), epoxomicin-treated 26S (Figure 3C), lactacystin-treated 26S (data not shown), or epoxomicin-treated cytosol (data not shown). Hence, the 26S protease activity was responsible for the different quenching profiles observed with 19S and 26S. This conclusion was confirmed by measuring the BOF intensity of intact and proteolyzed Δgpαf-BOF and its sensitivity to αBOF (Supplemental Data).

Since the spectral change observed in 26S-containing samples includes contributions from both retrotranslocation and degradation, the kinetics of retrotranslocation are best quantified using spectral changes that are independent of substrate digestion. Thus, the rate of Δgpαf-BOF exposure to the cytosol under our conditions is more accurately given by the t_1/2_ (the time required to reach 50% of maximal quenching) in 19S-containing samples (2.2 min; Figure 3C) than by the t_1/2_ in 26S-containing samples (8.3 min; Figure 3B). These data also reveal that the maximal initial rate of Δgpαf-BOF retrotranslocation can be achieved solely with ATP and PA700 (Figure 3D).

### Only PDI Is Required for Maximal Δgpαf Retrotranslocation

**Figure 4:**
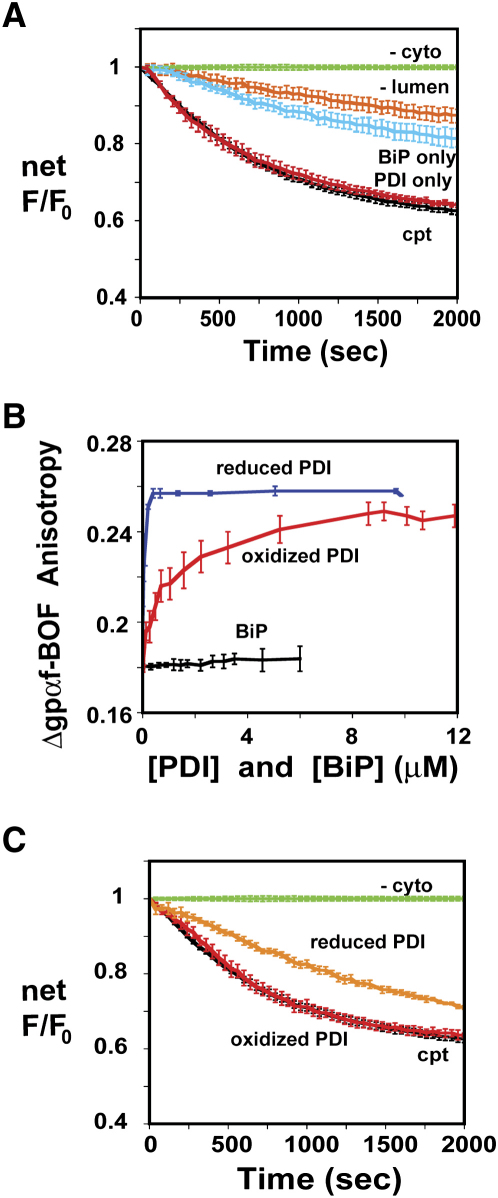
Retrotranslocation Dependence on Lumenal Components (A) RRMs containing Δgpαf-BOF and 2 mM ATP were reconstituted with total lumenal proteins (black), no lumenal proteins (orange, n = 5), BiP (cyan, n = 4), or PDI (red, n = 7). Each RRM was then incubated with cptcyto at 30°C. For comparison, cptRRMs incubated in the absence of all cytosolic proteins are shown (green). Net αBOF-dependent spectral changes are shown after subtraction of –cyto background (cf. Figure 2C). (B) Δgpαf-BOF (0.2 μM in buffer A) was titrated at 4°C with oxidized PDI (red, n = 6), reduced PDI (blue, n = 7), or purified BiP (black, n = 3). PDI was reduced or oxidized by 30 min incubation at 30°C in 5 mM DTT or 5 mM GSSG, respectively; BiP was incubated with 2 mM ATP for 10 min at 30°C. (C) RRMs containing Δgpαf-BOF and 2 mM ATP were reconstituted with total lumenal proteins (black), no cytosolic proteins (green), 10 μM oxidized PDI + 5 mM GSSG (red, n = 3), or 10 μM reduced PDI + 5 mM DTT (orange, n = 3). Data were analyzed as in (A).

To determine whether lumenal proteins are essential for Δgpαf-BOF retrotranslocation, RRMs were reconstituted with Δgpαf-BOF, ATP, and various combinations of lumenal proteins. When RRMs lacking all lumenal proteins were compared with cptRRMs, the net αBOF-dependent quenching was substantially reduced (Figure 4A). Thus, lumenal proteins are required for maximal Δgpαf-BOF retrotranslocation. However, some Δgpαf-BOF retrotranslocation occurred at a slow rate even in the absence of lumenal proteins.

To assess whether individual lumenal proteins stimulate retrotranslocation, RRMs containing only BiP, ATP, and Δgpαf-BOF in the lumen were incubated in cptcyto. BOF quenching was higher with these RRMs than with RRMs lacking all lumenal proteins (Figure 4A), which showed that BiP stimulated Δgpαf-BOF retrotranslocation. However, the extent of BiP stimulation was much less than that seen with a full complement of lumenal proteins and was not increased by a higher BiP concentration. In contrast, when only PDI, ATP, and Δgpαf-BOF were reconstituted into RRMs, the maximal rate and extent of BOF quenching were essentially equivalent to those obtained with RRMs containing a full complement of lumenal proteins (Figure 4A). Thus, only PDI is required in the lumen for maximal Δgpαf-BOF retrotranslocation.

### PDI Binds Directly to Δgpαf-BOF that Lacks Cys

PDI must stimulate retrotranslocation by interacting with the ER membrane and/or the substrate. Free Δgpαf-BOF was titrated with either reduced or oxidized PDI, and its binding to Δgpαf-BOF was detected by an increase in BOF anisotropy caused by the slower rotational rate of PDI•Δgpαf-BOF than of Δgpαf-BOF. Both oxidized and reduced PDI associated with Δgpαf (Figure 4B), but reduced PDI had a significantly higher affinity for this ERAD substrate than oxidized PDI did (K_d_ values of ∼30 and ∼900 nM, respectively). Thus, PDI bound tightly to Δgpαf-BOF even though it lacked disulfides and free thiols (see below). In contrast, BiP did not detectably bind to Δgpαf-BOF (Figure 4B).

Does the higher affinity of Δgpαf for reduced PDI than for oxidized PDI affect the rate of retrotranslocation? When Δgpαf-BOF was reconstituted into RRMs with ATP and either oxidized or reduced PDI, the rate of retrotranslocation was significantly lower with reduced PDI than with either oxidized (Figure 4C) or untreated (Figure 4A) PDI (PDI purified using our procedures is air oxidized; N.J.B., unpublished data). Thus, although the reduced form of PDI binds Δgpαf more tightly, maximal retrotranslocation rates require oxidized PDI and/or oxidation conditions in the lumen.

### Derlin-1 Is Essential for Δgpαf Retrotranslocation

ERAD substrate transport from the lumen to the cytosol was originally proposed to occur through the Sec61α translocon pore, but more recent evidence has implicated derlin-1 as a component of a retrotranslocation site (Lilley and Ploegh, 2004; Ye et al., 2004; Schekman, 2004). We therefore assessed the involvement of Sec61α and derlin in Δgpαf-BOF retrotranslocation using both fluorescence and photocrosslinking techniques.

**Figure 5:**
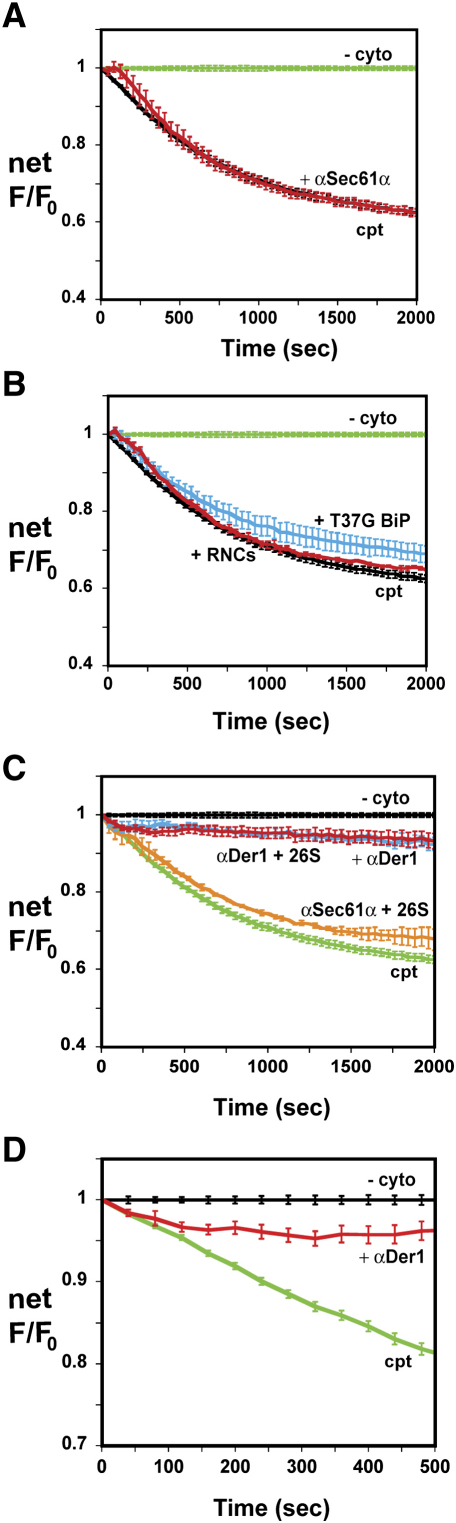
Retrotranslocation Dependence on Membrane Components (A and B) CptRRMs (∼10 Eq) were preincubated with 3 μg/ml αSec61α (red, n = 3) (A) or 10–20 nM RNCs, 40 nM SRP, and 1 mM GTP (red, n = 2) (B) before the addition of cptcyto and incubation at 30°C. Also in (B), RRMs containing 20 μM T37G BiP mutant and 2 mM ATP were incubated in cptcyto (cyan, n = 5). (C) CptRRMs were preincubated with 3 μg/ml αDer1 (red, n = 6; cyan, n = 3) or αSec61α (orange, n = 4) before the addition of cptcyto (red) or 20 μg/ml 26S proteasomes (cyan, orange) and incubation at 30°C. For comparison, cptRRMs incubated in the presence ([A] and [B] = black; [C] = green) or absence ([A] and [B] = green; [C] = black) of all cytosolic proteins are shown in (A)–(C). The αBOF-dependent quenching is shown after subtraction of –cyto background (cf. Figure 2C). (D) Data in (C) shown on an expanded scale.

We previously showed that affinity-purified antibodies against Sec61α (αSec61α) blocked iodide ion passage through the aqueous pores of ribosome-free translocons (Hamman et al., 1998). It was therefore possible that αSec61α could also inhibit Δgpαf-BOF retrotranslocation. Yet preincubating cptRRMs with αSec61α altered neither the rate nor the extent of Δgpαf-BOF quenching (Figure 5A).

The cytosolic end of the translocon pore can also be blocked by the binding of a ribosome (Crowley et al., 1994; Gillece et al., 2000; Schmitz et al., 2000). Although this blockage is not stoichiometric (∼50% of RRM translocons are bound under our conditions; Hamman et al., 1998), one might expect a 50% reduction in potential retrotranslocation channels to reduce the maximal rate of retrotranslocation. Ribosome-nascent chain complexes (RNCs) with an 86-residue preprolactin nascent chain were purified and then incubated with cptRRMs and SRP to target the RNCs to Sec61α translocons. The resulting RRMs were incubated with cptcyto to initiate retrotranslocation, and the rate of Δgpαf retrotranslocation was not affected by the tight binding of RNCs to translocons (Figure 5B).

The lumenal end of the aqueous translocon pore can be closed by BiP, either directly or indirectly (Hamman et al., 1998; Haigh and Johnson, 2002; Alder et al., 2005). Whereas BiP-mediated closure is regulated and reversible under normal conditions, the pores in ribosome-free translocons are closed irreversibly if RRMs are prepared with ATP and a T37G BiP mutant in which communication between the nucleotide and peptide-binding domains is defective (Alder et al., 2005). When RRMs containing T37G BiP, ATP, and Δgpαf-BOF were incubated with cptcyto, the rate and extent of αBOF-dependent quenching did not differ significantly from samples containing wild-type BiP (Figure 5B). These data strongly suggest that Δgpαf-BOF does not pass through the translocon pore during retrotranslocation.

In contrast, when cptRRMs were preincubated with αDer1 (affinity-purified antibodies that bind to the C-terminal region of derlin-1), αBOF-dependent quenching, and hence Δgpαf-BOF retrotranslocation, was reduced by more than 80% (Figure 5C). αDer1, but not αSec61α, also blocked 26S stimulation of retrotranslocation (Figure 5C). Yet the αDer1 effect was more complex than simple blockage: even after RRMs were incubated with an excess of αDer1 at 0°C, the rate of αBOF-dependent quenching was initially unaltered (Figure 5D). But after 2 min at 30°C, Δgpαf-BOF retrotranslocation was totally blocked. Thus, αDer1 can block the retrotranslocation machinery, but only after a short lag.

### Δgpαf Photocrosslinks Derlin-1

**Figure 6:**
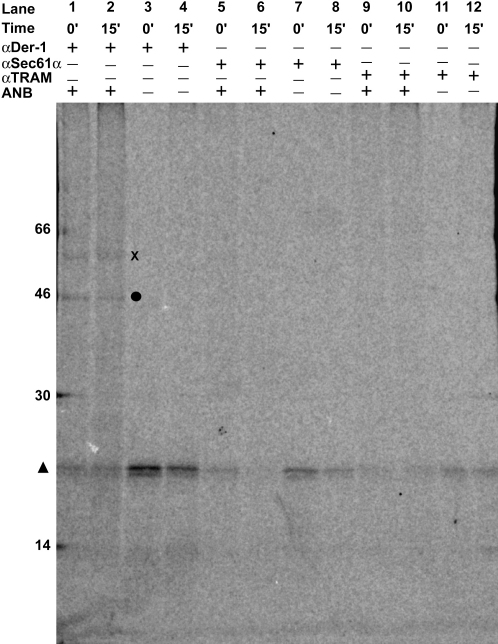
Δgpαf Photocrosslinking to Derlin-1 pΔgpαf mRNA was translated in the presence of either ɛANB-Lys-tRNA^Lys^ or Lys-tRNA^Lys^ as in Figure 1 and translocated into microsomes in vitro in the dark as indicated. Microsomes were purified and then photolyzed either before or after a 15 min incubation at 30°C. Photolyzed samples were split and immunoprecipitated with affinity-purified antibodies αDer1, αSec61α, or αTRAM as indicated. Δgpαf-derlin-1 photoadduct, •; Δgpαf, ▴; unidentified species also seen in nonimmune IgG control, X.

To determine which molecules are adjacent to Δgpαf during retrotranslocation, photoreactive probes were incorporated into pΔgpαf in an in vitro translation containing N^ɛ^-(5-azido-2-nitrobenzoyl)-Lys-tRNA^Lys^ (ɛANB-Lys-tRNA^Lys^; Krieg et al., 1986). After microsomes were pelleted and resuspended in cptcyto, the sample was halved and photolyzed either before or after retrotranslocation was initiated by raising the temperature to 30°C. After aliquots were removed to examine photoadduct formation in the total sample and in the microsome and supernatant fractions (Figure S4), the remainder was split into thirds and immunoprecipitated with antibodies to Der1, Sec61α, or TRAM, a core component of the translocon (Johnson and van Waes, 1999). A photoadduct was immunoprecipitated with αDer1 (Figure 6, lanes 1 and 2), but not with αSec61α (Figure 6, lanes 5 and 6) or αTRAM (Figure 6, lanes 9 and 10). No photoadducts were seen in the absence of probe (Figure 6, lanes 3 and 4). Since photolyzed ANB has a short reactive lifetime, photoadducts are formed only with molecules in close proximity to Δgpαf at the time of photolysis. Therefore, Δgpαf was adjacent to derlin-1 but not detectably proximal to Sec61α or TRAM.

The number of Δgpαf photocrosslinks to cytosolic proteins increased over time, thereby indicating that the photoreactive Δgpαf was transported through the retrotranslocation site during the 30 min incubation (Figure S4). Yet the yield of Δgpαf-derlin-1 photoadduct was approximately the same at 0°C and 30°C (Figure 6, lanes 1 and 2), which shows that Δgpαf was adjacent to derlin-1 both before and after retrotranslocation was initiated (see below). Since the photoreactive probes were incorporated randomly into the nine Lys codons of Δgpαf, not all probes in a single Δgpαf were adjacent to retrotranslocation-site proteins at the time of photolysis. Furthermore, only a few of the photoreactive Δgpαf molecules were in the retrotranslocation site at the time of photolysis. For these reasons, the yield of Δgpαf-derlin-1 photoadduct was relatively low. However, the critical observation is that Δgpαf photoadducts were formed with derlin-1, but not with Sec61α or TRAM.

## Discussion

The real-time continuous detection of Δgpαf-BOF retrotranslocation and its dependence on various components provides a unique perspective on the mechanisms and roles of individual proteins during retrotranslocation in mammals. This initial study revealed, among other things, that Δgpαf-BOF retrotranslocation occurred rapidly and was complete within minutes, required both ATP and the proteasome, was ubiquitin independent, was p97 and cofactor independent, and occurred at the same rate with total cytosolic proteins, purified 26S proteasomes, or purified PA700. In addition, the rate of Δgpαf-BOF retrotranslocation was maximal with either the full complement of lumenal proteins or with oxidized PDI, was decreased in the absence of all soluble lumenal proteins, was increased only slightly by purified BiP and ATP, and was decreased in the presence of reduced PDI. We also discovered that αDer1 rapidly blocked Δgpαf-BOF efflux, that Δgpαf photocrosslinked to derlin-1 but not to Sec61α, that neither αSec61α nor RNCs inhibited Δgpαf-BOF retrotranslocation, and that a mutant BiP that irreversibly closes the lumenal end of the translocon pore had no effect on retrotranslocation. Since all experiments were performed with intact membranes, these combined photocrosslinking and fluorescence data constitute the most direct evidence to date that derlin-1 is located in close proximity to the pore through which Δgpαf moves through the ER membrane.

The fluorescence approach introduced here characterizes retrotranslocation with unprecedented time resolution. ERAD substrate movement from the lumen to the cytosol is detected directly and in real time, thereby allowing both the kinetics and the extent of retrotranslocation to be reproducibly quantified and compared. Since this spectral approach is nondestructive, substrate environment can be monitored continuously. More importantly, this approach examines substrate retrotranslocation under native conditions. Since membranes and macromolecular complexes are intact throughout, the functional effects of even weak or transient interactions or assemblies can be detected without being disrupted by the detergent required for some analyses. Furthermore, since different aspects of the fluorescence signal can be monitored (e.g., intensity, anisotropy, lifetime, fluorescence resonance energy transfer, and accessibility to quenchers; Johnson, 2005), spectral changes can simultaneously monitor both retrotranslocation and other alterations in substrate environment and conformation (e.g., proteolysis [Figure 3C] and association with PDI [Figure 4B]). The ability to resolve different intermediate states and interactions of the substrate allows mechanistic questions to be addressed at a higher resolution. For example, spectroscopically differentiating between Δgpαf-BOF exposure to and degradation in the cytosol (Figure 3C) shows that retrotranslocation precedes substrate proteolysis, as expected, and that the actual rate of retrotranslocation is best determined using fluorescence rather than proteolysis assays.

We must emphasize that we have only characterized the retrotranslocation of a single ERAD substrate in this initial study, and other substrates may require other components or pathways for retrotranslocation. For example, certain lumenal factors appear to be involved in the retrotranslocation of glycosylated ERAD substrates (Hebert et al., 2005). But the experimental approach described here can, in principle, be used with any soluble ERAD substrate or derivative. The biochemical reconstitution of samples provides great flexibility in examining the influence of specific soluble proteins or small molecules on either side of the membrane on the rate and extent of retrotranslocation. Not only are the samples homogeneous and well defined in terms of components and their concentrations, the substrates are in a uniform environment and conformation inside the lumen. Deleting a wild-type protein or replacing it with a purified mutant protein during reconstitution, as was done here, provides the same flexibility in a mammalian system that is achieved genetically in yeast.

Several aspects of our data were unexpected. BiP has been shown in previous studies to be required for the retrotranslocation of several substrates in yeast (Nishikawa et al., 2005), including Δgpαf (Brodsky et al., 1999; Kabani et al., 2003), yet it had only a small stimulatory effect on Δgpαf retrotranslocation from mammalian microsomes (Figure 4A). Also, although BiP can function as a chaperone, it appears to bind weakly, if at all, to Δgpαf (Figure 4B). BiP's role in retrotranslocation may therefore be more complex than is currently appreciated. In contrast, only oxidized PDI was required in the lumen to obtain maximal retrotranslocation (Figures 4A and 4C). The mechanism by which PDI promotes retrotranslocation is not yet understood but may involve PDI binding to the ERAD substrate (Gillece et al., 1999; Tsai et al., 2001). Since PDI promotes the folding of proteins that lack disulfide bonds and unreacted Cys (Puig and Gilbert, 1994; Cai et al., 1994; Wilkinson and Gilbert, 2004), it was not surprising that PDI bound Δgpαf (Figure 4B). However, the lower affinity of Δgpαf for oxidized PDI than for reduced PDI was unexpected because retrotranslocation was faster with oxidized PDI than with reduced PDI (Figure 4C). This result suggests that tight binding of PDI to Δgpαf inhibits retrotranslocation. It is also possible that oxidized, but not reduced, PDI interacts with the retrotranslocation machinery at the membrane and/or that the presence of 5 mM DTT in the microsomes reduces lumenal disulfide bonds and slows retrotranslocation. But since cholera toxin movement from the lumen to the cytosol is promoted by reducing agents (Tsai et al., 2001), PDI involvement in retrotranslocation is likely substrate dependent.

The presence of p97, Npl4, and Ufd1 in the cytosol, either by themselves or added to total cytosolic proteins, did not stimulate Δgpαf retrotranslocation (Figure 3A). Since these proteins facilitate the retrotranslocation of most examined ubiquitinylated substrates (Bays and Hampton, 2002; Tsai et al., 2002) and Δgpαf is not ubiquitinylated, this result may be expected. But it shows, contrary to some suggestions, that p97 is not required for the retrotranslocation of every ERAD substrate (Lee et al., 2004; Kothe et al., 2005; Wojcik et al., 2006; Carlson et al., 2006). Instead, PA700 is sufficient for maximal Δgpαf retrotranslocation (Figures 3B and 3D). Since ATP is also required and NEM-treated PA700 was retrotranslocation defective, it seems likely that AAA ATPase components of PA700 are involved in powering substrate movement through the membrane via the chaperone-like properties of PA700 (Strickland et al., 2000; Liu et al., 2002). The roles of individual PA700 subunits in retrotranslocation will be clarified by future experiments.

The membrane proteins that form the channel through which ERAD substrates are transported from the lumen to the cytosol have not been identified. Retrotranslocation was initially proposed to occur via the Sec61α translocon pore (Wiertz et al., 1996; Plemper et al., 1997; Pilon et al., 1997; Zhou and Schekman, 1999), and some subsequent data are consistent with this model (Gillece et al., 2000; Schmitz et al., 2000; Lee et al., 2004; Kalies et al., 2005; Ng et al., 2007). Other studies have proposed that derlin-1 and its homologs, derlin-2 and derlin-3, are located at the site of retrotranslocation (Ye et al., 2005; Lilley and Ploegh, 2005; Oda et al., 2006; Lilley et al., 2006). As summarized above, we found in this study that the rate and extent of Δgpαf-BOF retrotranslocation were not affected by closing either end of the translocon pore. Instead, two independent techniques—Δgpαf photocrosslinking to derlin-1 (Figure 6) and αDer1 blockage of Δgpαf-BOF retrotranslocation (Figure 5D)—strongly indicated that derlin-1 is located at the site of retrotranslocation. Our data therefore strongly suggest that derlin-1 is a structural and/or functional component of the site of Δgpαf retrotranslocation. Future experiments will determine whether or not more complex ERAD substrates are retrotranslocated through different retrotranslocation sites, molecular assemblies, and/or pathways.

Continuous monitoring of retrotranslocation revealed that αDer1 did not block Δgpαf retrotranslocation at t_0_ but did block retrotranslocation after 2 min (Figure 5D). The delay presumably resulted from αDer1 being initially unable to bind to derlin-1 or block its function when the antibodies were mixed with the RRMs. One possible explanation is that αDer1 does not bind to a derlin-1 that is already engaged with an ERAD substrate but does bind to an unoccupied derlin-1. In this case, any Δgpαf-BOF bound to the retrotranslocation site at 0°C would be transported normally to give the maximal initial rate of retrotranslocation, but further retrotranslocation would be blocked when αDer1 bound to retrotranslocation sites following substrate release. Since Δgpαf photocrosslinking to derlin-1 was the same at t_0_ and at t = 15 min (Figure 6), Δgpαf was positioned adjacent to derlin-1 at both 0°C and 30°C. These combined data suggest that Δgpαf can be trapped at an intermediate stage of retrotranslocation by varying the temperature: Δgpαf appears to be targeted to a derlin-1-containing retrotranslocation site at low temperature to form a membrane-bound intermediate and only passes through the site when the temperature is raised (Figure 5D; Figure 6; Figure S3). If so, the C-terminal end of Δgpαf must move through the retrotranslocation site in <2 min (Figure 5D). Future real-time spectroscopic characterizations of retrotranslocation using various substrates and the mammalian biochemical approach described here will provide additional insights into the mechanisms that regulate and accomplish retrotranslocation.

## Experimental Procedures

### Reconstituted Rough Microsomes

Canine ER microsomes were extracted by exposure to high pH and then reconstituted on ice with 4–5 μM Δgpαf-BOF, 2 mM ATP, and 3.5 mg/ml of total soluble lumenal proteins to form cptRRMs (Alder et al., 2005). Alternatively, RRMs were prepared with 2 mM ATP and either 10–20 μM BiP or 10–20 μM PDI in the presence of either 5 mM DTT or 5 mM GSSG. For some experiments, extracted RRMs were incubated (0°C, 5–10 min) with 20 μM T37G BiP + 2 mM ATP before Δgpαf-BOF and other lumenal proteins were added to complete the reconstitution. Before use, RRMs were gel filtered (Sepharose CL-2B; 50 cm × 0.7 cm inside diameter) at 4°C to remove nonencapsulated proteins.

### Retrotranslocation by Proteolysis

pΔgpαf mRNA was transcribed in vitro using SP6 RNA polymerase and then translated (350 μl, 26°C, 60 min) in a wheat-germ cell-free extract as before (Flanagan et al., 2003) in the presence of 150 μCi [^35^S]Met, 40 nM canine SRP, 96 equivalents (Eq) of salt-washed canine ER microsomes, and 210 pmol of *E. coli* Lys-tRNA^Lys^ or ɛBOF-Lys-tRNA^Lys^ (Woolhead et al., 2004). Microsomes were pelleted and resuspended in 350 μl of buffer B (2 mM ATP, 2 mM GTP, 16 mM phosphocreatine, 16 units/ml creatine phosphokinase, and protease inhibitors in buffer A [50 mM HEPES (pH 7.5), 40 mM KOAc, 5 mM MgCl_2_]) containing either 60 μl of reticulocyte lysate, 240 μl of cyto, or no lysate. Samples were then incubated at 30°C, and 100 μl aliquots were removed at the indicated times for analysis by SDS-PAGE on a 10%–20% gel. ^35^S and BOF fluorescence were quantified using a Bio-Rad FX imager.

### Retrotranslocation by Fluorescence

A photon-counting steady-state SLM 8100 with a double-grating excitation monochromator, a single-grating emission monochromator, and a 450 W xenon lamp was used for all experiments, as were 4 × 4 mm quartz cuvettes. (The same results were obtained with 1 × 1 cm cuvettes.) λ_ex_ and λ_em_ were 490 nm and 513 nm (4 nm band pass).

A retrotranslocation sample contained ∼10 Eq of RRMs that were incubated (0°C, 30 min) with an excess (30 μg/ml) of αBOF in 240 μl of buffer B; a parallel sample lacked αBOF. Because RRMs scatter light efficiently, two additional control samples were prepared that contained Δgpαf instead of Δgpαf-BOF. Each sample was then mixed with cyto (to a final concentration of 0.3 mg/ml; ±epoxomicin); 26S proteasomes (to 20 μg/ml; ±epoxomicin); PA700/19S RP (to 40 μg/ml; ±NEM); and/or p97, Npl4, and Ufd1 (to 0.25 μM each) to form a 300 μl final sample in buffer B. RRMs were equalized in samples using light scattering (λ_ex_ = 405 nm, λ_em_ = 420 nm, 4 nm band pass). Retrotranslocation was initiated at t_0_ by transferring the sample from ice to the SLM cell holder at 30°C. Emission intensities were recorded for 5 s at 40 s intervals. The intensities of the Δgpαf control samples did not change with time and were subtracted from the intensities of the equivalent Δgpαf-BOF samples to yield the net emission intensities. The αBOF-dependent intensity change (F) was given by the ratio of the net intensities measured at each time point in the presence versus absence of αBOF (F = F_+αBOF_/F_−αBOF_). The average F values and standard error of the mean for n independent experiments are shown in Figures 2–5.

In some experiments, RRMs were incubated (0°C, 30 min) with αSec61α (3 μg/ml) or αDer1 (3 μg/ml) prior to the addition of cytosolic proteins to form retrotranslocation samples (Figures 5B and 5C). In others, RNCs with an 86-residue preprolactin nascent chain were prepared using truncated mRNAs and purified as before (Flanagan et al., 2003). RNCs (10–20 nM) were then incubated (0°C, 30 min) with 10 Eq of cptRRMs and 40 nM SRP in buffer A + 2 mM GTP before mixing with cytosolic proteins in buffer B to initiate retrotranslocation.

### PDI Binding to Δgpαf-BOF

Δgpαf-BOF was titrated with BiP or PDI at 4°C in buffer A, and association was detected by an increase in anisotropy (r, measured as described previously; Johnson et al., 1982). Procedures were as described (Flanagan et al., 2003), except that a separate sample was prepared for each PDI or BiP concentration. Protein adsorption to cuvette walls was prevented by coating them as described (Ye et al., 1991).

### Photocrosslinking

pΔgpαf mRNA was translated as above (350 μl, 26°C, 60 min) in the dark, except that samples contained either 210 pmol of Lys-tRNA^Lys^ or 420 pmol of ɛANB-Lys-tRNA^Lys^. After microsomes were purified by sedimentation and mixed with cptcyto, aliquots (50 μl) were photolyzed either before (0 min sample) or after a 15 min incubation at 30°C. After pelleting, photoadducts were immunoprecipitated with αSec61α, αTRAM, or αDer1 as before (McCormick et al., 2003), except that αDer1 was bound in 140 mM NaCl, 10 mM Tris-HCl (pH 7.6), 1% (v/v) Triton X-100.

## Supplemental Data

Document S1. Supplemental Results, Supplemental Experimental Procedures, Supplemental References, and Four Figures
