# Supplementary material for: Real-Time Fluorescence Detection of ERAD Substrate Retrotranslocation in a Mammalian In Vitro System
Source: Cell. 2007 Jun 1;129(5):943–55. doi: 10.1016/j.cell.2007.03.046 (PMC1890003; doi:10.1016/j.cell.2007.03.046)
Supplement: Document S1. Supplemental Results, Supplemental Experimental Procedures, Supplemental References, and Four Figures [file mmc1.pdf]

Cell, Volume *129*

Supplemental Data

Real-Time Fluorescence Detection of ERAD Substrate Retrotranslocation in a Mammalian In Vitro System

Judit Wahlman, George N. DeMartino, William R. Skach, Neil J. Bulleid, Jeffrey L. Brodsky, and Arthur E. Johnson

Supplemental Results

Proteolysis and Fluorescence of Δgpαf-BOF

When cptRRMs were incubated in cptcyto at 30°C, the retrotranslocated Δgpαf-BOF was degraded (Figure S1A). Since this proteolysis was prevented by preincubating the cytosol with the proteasome-specific inhibitor lactacystin (Figure S1A), Δgpαf-BOF was degraded solely or primarily by the 26S proteasome. Biophysical experiments also showed that full-length non-ubiquitinated Δgpαf-BOF was degraded by purified 26S proteasomes, but not by purified epoxomicin-inhibited 26S proteasomes (Figure S1B). The reduction in BOF anisotropy (r) results from an increase in the dye's rate of rotation as the polypeptide to which the dye is attached is reduced in size by proteolysis. The same anisotropy changes were observed when BOF anisotropy was monitored in samples containing cptcyto ± epoxomicin (not shown), or when Δgpαf-BOF was proteolyzed with trypsin (not shown). Upon proteolysis of Δgpαf-BOF by purified 26S, BOF emission intensity was increased by 65%. Thus, BOF emission is quenched by 40% in intact Δgpαf.

αBOF Quenching of Intact and Proteolyzed Δgpαf-BOF

BOF emission is quenched upon binding to BOF-specific antibodies, and the intensity of the quenched αBOF-bound dye is the same whether BOF is attached to intact or degraded Δgpαf. Thus, the magnitude of observed antibody quenching depends upon the initial state of the Δgpαf polypeptide. When excess αBOF binds to intact Δgpαf-BOF, BOF intensity is reduced by 56%. When excess αBOF is added to proteolyzed Δgpαf-BOF, BOF intensity was reduced by 72%. Hence, when Δgpαf-BOF is being actively degraded in a sample, αBOF-dependent quenching ranges between 56% and 72%. Since αBOF binding to either intact or degraded Δgpαf-BOF is complete by the time the sample has been mixed, no detectable delay for αBOF binding to Δgpαf-BOF is observed, and no correction of the kinetic data is necessary.

The difference in αBOF quenching of intact and degraded Δgpαf-BOF explains why the observed spectral change was greater (net F/F_0 was smaller) in samples containing 26S proteasomes than in samples containing 19S proteasomal regulatory particles (RPs) (Figure 3B,C): $F_{-\alpha BOF}$ was greater in samples in which Δgpαf-BOF was degraded. Thus, there are five components to the spectral signal in our experiments: the Δgpαf-BOF molecules that were not retrotranslocated and hence not quenched; the Δgpαf-BOF molecules that were retrotranslocated and thereby exposed to αBOF; the Δgpαf-BOF molecules that were exposed to αBOF because of background microsome release during the incubation at 30°C (see below);

the Δgpαf-BOF molecules that were degraded in the cytosol; and the few Δgpαf-BOF molecules that were adsorbed to the outer surface of the microsomes after gel filtration (<10%). Clearly, it is essential to identify, quantify, and thoroughly characterize the biochemical origin of any spectral changes before interpreting them (Johnson, 2005).

The fraction of Δgpαf-BOF that is retrotranslocated from RRMs in our samples can be calculated using the above intensities for different species. Assuming that 10% of the Δgpαf-BOF is adsorbed to the outer surface of the microsomes and that 10% of the remaining Δgpαf-BOF (= 9%) is exposed to the cytosol by the background release of contents from microsomes (Figure S2A), a total intensity decrease of 46% (Figure 2C) in cptRRM + cptcyto samples under our conditions would result from 36% of the originally encapsulated Δgpαf-BOF being retrotranslocated from the lumen to the cytosol and then digested within 2000 sec. As noted in the text, this magnitude of retrotranslocation is similar to the fraction of Δgpαf-BOF found in the cytosol after the microsomes have been sedimented (Figure 2D).

Origin of Background Fluorescence Quenching

Is the slow, steady increase in αBOF-dependent quenching in the absence of cytosolic proteins or ATP (Figure 2B) caused by encapsulated material leaking from the microsomes? RRMs were reconstituted with ATP, total lumenal proteins, and BOF-labeled glutathione to determine if the release of a small molecule occurs spontaneously from the RRMs over time. Other RRMs were prepared with ATP and either BOF-labeled BiP or PDI because these proteins are located in the ER lumen and should not be substrates for retrotranslocation. When incubated in cptcyto, the rates and extents of quenching, and hence BOF exposure to αBOF in the cytosol, were the same for both large (BiP-BOF, PDI-BOF) and small (glutathione-BOF) encapsulated molecules (Figure S2A). Thus, any openings in the RRMs had to be large enough to release PDI and BiP at the same rate as glutathione. Yet we showed previously that microsomal membranes are impermeable to iodide ions for more than 4 hours at 4°C (Crowley et al., 1994). Thus, holes large enough to release PDI or BiP from microsomes are unlikely, and the slow increase in αBOF-dependent quenching in the absence of retrotranslocation apparently does not occur due to either glutathione-BOF or Δgpαf-BOF leakage from RRMs.

Instead, this signal loss most likely results from a low constant rate of RRM breakage at 30°C that simultaneously exposes glutathione-BPF and the larger Δgpαf-BOF, PDI-BOF, and BiP-BOF to αBOF. Whatever its origin, this αBOF-dependent emission intensity decrease appears to constitute a "background" signal change because it is observed under conditions in which retrotranslocation does not occur (compare Figure 2B "–cyto" and "–ATP" traces with Figure S2A). Moreover, after very long time periods (~50 min), the rate of αBOF-dependent Δgpαf-BOF quenching was the same in the presence and absence of cytosolic proteins. Thus, only background quenching was observed at long times (Figure S2B). The quenching due to retrotranslocation was complete within ~50 min under our conditions in the sample containing cytosolic proteins, and the *net* intensity reach a plateau by 50 min (Figure 2C). We have therefore routinely subtracted the αBOF-dependent quenching observed with samples lacking cytosol to accurately portray the retrotranslocation-dependent fluorescence change.

Temperature Effects

CptRRMs were incubated in parallel with or without cytosolic proteins at different temperatures. Since the –cyto background quenching was temperature-dependent, the –cyto signal was subtracted from the +cyto signal to yield the net αBOF-dependent quenching at each temperature (Figure S3). These data revealed that both the rate and extent of mammalian retrotranslocation are temperature dependent under our conditions. No retrotranslocation was detected at 4°C after 1 hr. Hence, no retrotranslocation occurs while our samples are on ice prior to raising their temperature at t_0 to initiate retrotranslocation. The net αBOF-dependent

quenching (retrotranslocation) was essentially the same at 37°C and 30°C (Figure S3). Thus, our experiments were done at 30°C because the extent of non-retrotranslocation release of Δgpαf-BOF, apparently by microsomal rupture, was lower.

Photocrosslinking of Lumenal, Cytosolic, and Membrane Components

Microsomes containing photoreactive [^{35}S]Δgpαf were incubated in cptcyto at 30°C for 0, 15, or 30 min (Experimental Procedures; Figure 6). At each time point, the radioactive protein species in the total sample were detected using SDS-PAGE (Fig, 4A). Following sedimentation to separate the microsomal pellet from the soluble protein supernatant, the radioactive species in the cytosol (Figure 4B) and in the microsomes (Figure 4C) were visualized by SDS-PAGE. Even prior to the 30°C initiation of retrotranslocation, many membrane-bound photoadducts with apparent molecular masses that exceed that of Δgpαf are visible (Figure 4C, lanes 1,4). The intensities of these bands are not greatly altered at later times (Figure 4C, lanes 2, 3, 5, 6). In contrast, the extent of Δgpαf photocrosslinking to cytosolic proteins increases significantly as retrotranslocation proceeds and the number of photoreactive Δgpαf molecules transported into the cytosol increases (Figure 4B, lanes 4-6). While likely photocrosslinking targets can be identified based on the apparent masses of the expected photoadducts, it is clear from the multiplicity of bands that the extent of Δgpαf photocrosslinking to Derlin-1, Sec61α, TRAM, and other membrane, lumenal, and cytosolic proteins is best determined by immunoprecipitation.

Supplemental Experimental Procedures

Proteins

Hemin-free rabbit reticulocyte lysate was prepared as described (Carlson et al., 2005), and hemoglobin was removed by passing the lysate through HisTrap HP resin (General Electric) in Buffer A [50 mM HEPES (pH 7.5), 40 mM KOAc, 5 mM $MgCl_2$] at 4°C, adding EDTA (pH 7.5) to 1 mM, and dialyzing overnight at 4°C against Buffer A + 4 mM reduced glutathione. Bovine 26S proteasomes and PA700 were purified as detailed elsewhere (DeMartino et al., 1994; Liu et al., 2006). p97, Npl4, and Ufd1 were purified as a complex (GND, in preparation). To inhibit proteolysis, 60 μM epoxomicin (Calbiochem) was incubated (0°C, 30 min) with 26S proteasomes or cyto. ATPase activity of PA700 was inactivated by incubation with N-ethylmaleimide (NEM) as described previously (DeMartino et al., 1994). Human intein-tagged PDI was purified using a chitin column according to Novagen specifications, while hamster BiP with C-terminal His tags was purified as before (Alder et al., 2005). PDI and BiP were each then additionally purified on a Q-Sepharose (GE Healthcare) column using a linear salt gradient. BiP, PDI, and Δgpαf are each stored in 50 mM HEPES (pH 8.0), 250 mM sucrose**;** the BiP solution also contained 1 mM ATP. Before use, PDI was usually incubated at 30°C for 30 min in Buffer A containing either 5 mM DTT or 5 mM G-S-S-G. αBOF was purchased from Invitrogen, affinity-purified αTRAM and αSec61α from Research Genetics (Huntsville, AL), and affinity-purified αDer1 from Novus Biologicals (Littleton, CO). Canine SRP and salt-washed ER microsomes were prepared as before (Flanagan et al., 2003; Walter and Blobel, 1983a; Walter and Blobel, 1983b).

Δgpαf-BOF

The following alterations were made in the wild-type S. cerevisiae pαf sequence: (i) N23Q, N57Q, and N67Q mutations prevented glycosylation; (ii) Y165C permitted the site-specific attachment of a single fluorescent dye; and (iii) a hexameric histidine tag at the C-terminus facilitated purification. No signal sequence was present because the protein was encapsulated

into RRMs biochemically, not via SRP. The primary sequence of Δgpαf was confirmed by DNA sequencing of the plasmid. The DNA encoding Δgpαf was cloned into a heat-inducible expression vector (Bush et al., 1991) and Δgpαf was overexpressed in *E. coli* BL21 (DE3) cells. After cell lysis, Δgpαf was bound to an XK 16/10 Chelating Sepharose Fast Flow column (GE Healthcare) loaded with Co^{2+}, equilibrated in Buffer C [8 M urea, 50 mM HEPES (pH 8.0), 100 mM NaCl], and eluted at 3 ml/min with 300 mM imidazole, 50 mM HEPES (pH 8.0).

The Δgpαf solvent was changed to Buffer D [50 mM Hepes (pH 8.0), 2 mM EDTA, 1 mM dithiothreitol (DTT), 2 M urea] by gel filtration through Sephadex G-25 (30 cm x 2.5 cm i.d.). Δgpαf was further purified by ion exchange chromatography on FPLC Q-Sepharose using a linear salt gradient (100-1000 mM NaCl in Buffer D without urea); Δgpαf eluted in a single peak near 300 mM NaCl. After Δgpαf was transferred into 20 mM Hepes (pH 8.0), 50 mM NaCl, and 2 mM EDTA by gel filtration to remove DTT prior to labeling, a 4-fold molar excess of 4,4-difluoro-5,7-dimethyl-4-bora-3a,4a-diaza-*S*-indacene-3-propionyl)-*N*'-iodoacetylethylenediamine (Invitrogen) dissolved in DMSO was added dropwise to Δgpαf (10-20 μM; ~50 ml) at 4°C with stirring (final DMSO concentration ≤ 3% v/v). After at least 12 hr in the dark at 4°C,the reaction was quenched (30 min, 4°C) by the addition of 10 mM DTT. Unreacted dyes were removed by first loading a volume of Buffer C equal to the protein reaction volume on a Sephadex G-25 gel filtration column (25 cm x 2.5 cm i.d.) that had been equilibrated in 50 mM HEPES (8.0), 250 mM sucrose. After the Buffer C had fully entered the resin, the reaction mix was loaded on the column so that the protein was eluted through the urea-containing buffer C, thereby releasing any non-covalently bound dye before the protein was finally eluted in 50 mM HEPES (8.0), 250 mM sucrose. The resulting Δgpαf-BOF (5-15 μM) was stored at –80°C in aliquots appropriate for reconstitution. No non-covalently bound BODIPY dyes were detected in Δgpαf-BOF preparations after SDS-PAGE electrophoresis and fluorescence detection using the Bio-Rad FX imager.

Gel filtration was used throughout to exchange the solvent because: (i) diluting the protein reduced aggregation; (ii) the BOF labeling efficiency was higher with gel-filtered Δgpαf than with dialyzed Δgpαf; and (iii) the labeled protein aggregated during a long dialysis at high concentration. Protein aggregation was also a problem when the pH was lowered below 7.5.

When examined by analytical HPLC using a C1 column to separate unlabeled and BOF-labeled Δgpαf proteins, 48-50% of the Δgpαf was found to be labeled with BOF using the above conditions. Since purifying Δgpαf-BOF from Δgpαf proved difficult, the Δgpαf-BOF used here was only about half-labeled.

BOF Labeling of BiP, PDI, and Glutathione

BiP and PDI were reacted with BOF using the same procedures used for labeling Δgpαf and were purified by gel filtration from the unreacted dye. Reduced glutathione (1 mM) was reacted with 10 μM BOF reagent; unreacted glutathione was not separated from glutathione-BOF.

RRMs

For the control experiments shown in Figure S2A, RRMs were prepared with 4-5 μM BiP-BOF, 4-5 μM PDI-BOF, or 10 μM glutathione-BOF instead of Δgpαf-BOF (Alder et al., 2005).

Preparation of Lumenal Proteins

β-Octylglucoside (0.4 M) was added drop-wise to a final concentration of 20 mM to 20 ml of 2 Eq/μl of salt-washed canine pancreatic microsomes with gentle stirring on ice. [1 Eq/μl of rough microsomes (RMs) has an absorbance of 50 A_{280} units/μl (Walter and Blobel, 1983a).] Ten ml of detergent-saturated membranes were layered on a 10-ml 50 mM Hepes (pH 7.5), 0.5 M sucrose cushion and sedimented (Ti 50.2, 40,000 rpm, 2 hr, 4°C). The supernatant fraction and the

upper 2 ml of the sucrose cushion (22 ml) were removed and re-sedimented (Ti 50.2, 40,000 rpm, 5 hr, 4°C). Detergent was removed from the second supernatant and upper 2 ml of the sucrose cushion by extensive dialysis (buffer exchanges occurred after 3, 14, 4, and 4 hr) at 4°C against 1 L of Buffer A without DTT. SM-2 Bio-beads (5 g; BioRad) were added to the dialysate for the first two dialyses, while the protein was equilibrated against 50 mM HEPES (pH 7.5), 250 mM sucrose during the last two 4-hr dialyses. After sedimentation (TLA100.2, 100,000 rpm, 30 min, 4°C), the supernatant (22 ml) containing all of the lumenal proteins was concentrated to 4 ml of 20 mg/ml using Centricon YM-3 filters (Amicon) before storage at –80°C in 50-μl aliquots.

Supplemental References

Alder, N. N., Shen, Y., Brodsky, J. L., Hendershot, L. M., and Johnson, A. E. (2005). The Molecular Mechanisms Underlying BiP-Mediated Gating of the Sec61 Translocon of the Endoplasmic Reticulum. J Cell Biol *168*, 389-399.

Bush, G. L., Tassin, A.-M., Fridén, H., and Meyer, D. I. (1991). Secretion in yeast. Purificaton and *in vitro* translocation of chemical amounts of prepro-α-factor. J Biol Chem *266*, 13811-13814.

Carlson, E., Bays, N., David, L., and Skach, W. R. (2005). Reticulocyte lysate as model system to study ER membrane protein degradation. Methods Mol Biol *301*, 185-205.

Crowley, K. S., Liao, S., Worrell, V. E., Reinhart, G. D., and Johnson, A. E. (1994). Secretory proteins move through the endoplasmic reticulum membrane via an aqueous, gated pore. Cell *78*, 461-471.

DeMartino, G. N., Moomaw, C. R., Zagnitko, O. P., Proske, R. J., Chu-Ping, M., Afendis, S. J., Swaffield, J. C., and Slaughter, C. A. (1994). PA700, an ATP-dependent activator of the 20 S proteasome, is an ATPase containing multiple members of a nucleotide-binding protein family. J Biol Chem *269*, 20878-20884.

Flanagan, J. J., Chen, J.-C., Miao, Y., Shao, Y., Lin, J., Bock, P. E., and Johnson, A. E. (2003). Signal Recognition Particle Binds to Ribosome-bound Signal Sequences with Fluorescence-detected Subnanomolar Affinity That Does Not Diminish as the Nascent Chain Lengthens. J Biol Chem *278*, 18628-18637.

Johnson, A. E. (2005). Fluorescence Approaches for Determining Protein Conformations, Interactions, and Mechanisms at Membranes. Traffic *6*, 1078-1092.

Liu, C.-W., Li, X., Thompson, D., Wooding, K., Chang, T.-l., Tang, Z., Yu, H., Thomas, P. J., and DeMartino, G. N. (2006). ATP binding and ATP hydrolysis play distinct roles in the function of 26S proteasome. Mol Cell *24*, 39-50.

Walter, P., and Blobel, G. (1983a). Preparation of microsomal membranes for cotranslational protein translocation. Methods Enzymol *96*, 84-93.

Walter, P., and Blobel, G. (1983b). Signal recognition particle: A ribonucleoprotein required for cotranslational translocation of proteins, isolation and properties. Methods Enzymol *96*, 682-691.

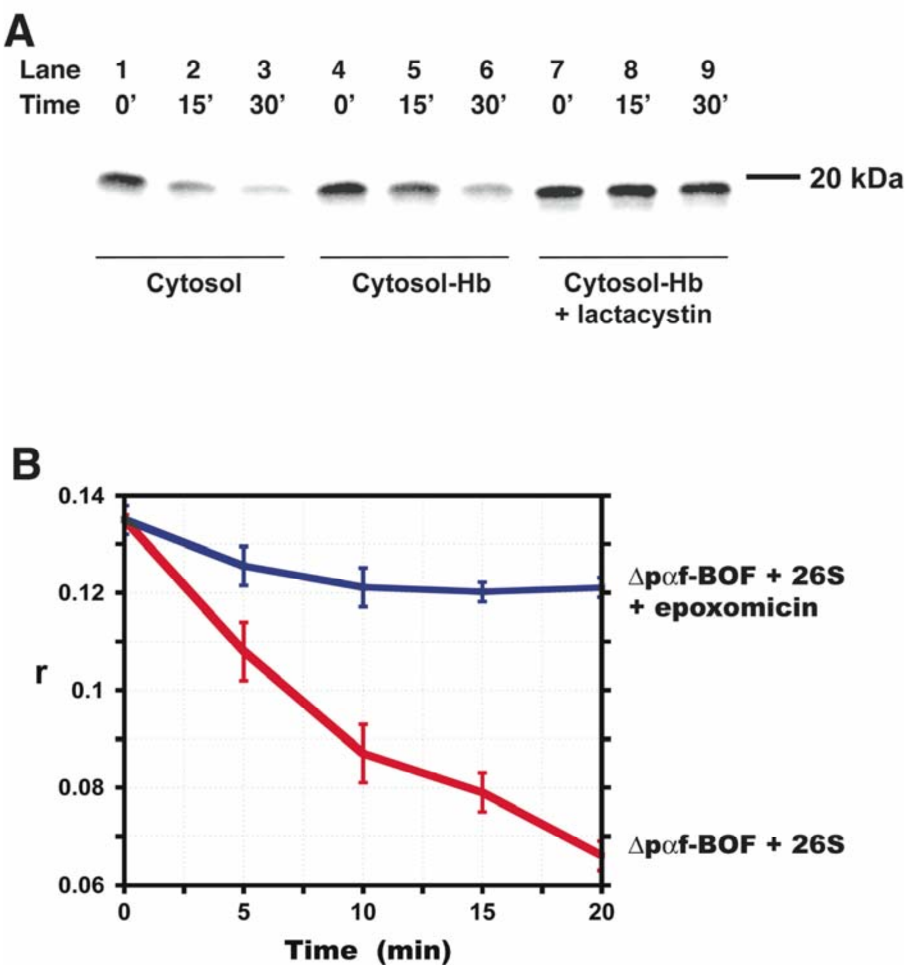

Figure S1. Fluorescence-Detected Proteolysis of Δgpαf-BOF

(A) CptRRMs were incubated at 30°C in either rabbit reticulocyte lysate (cytosol; lanes 1-3), cptcyto (cytosol–Hb; lanes 4-6), or cptcyto preincubated (0°C, 30 min) with 50 μM lactacystin (lanes 7-9). Equivalent aliquots of the total sample were removed at the times indicated and analyzed by SDS-PAGE. Full-length Δgpαf-BOF was detected by BOF emission intensity.

(B) The average (n = 3) anisotropy (r) of 0.5 μM purified Δgpαf-BOF was monitored as a function of time at 30°C in samples containing 20 μg/ml purified 26S proteasomes that had not (red) or had (blue) been preincubated (0°C, 30 min) with 60 μM epoxomicin.

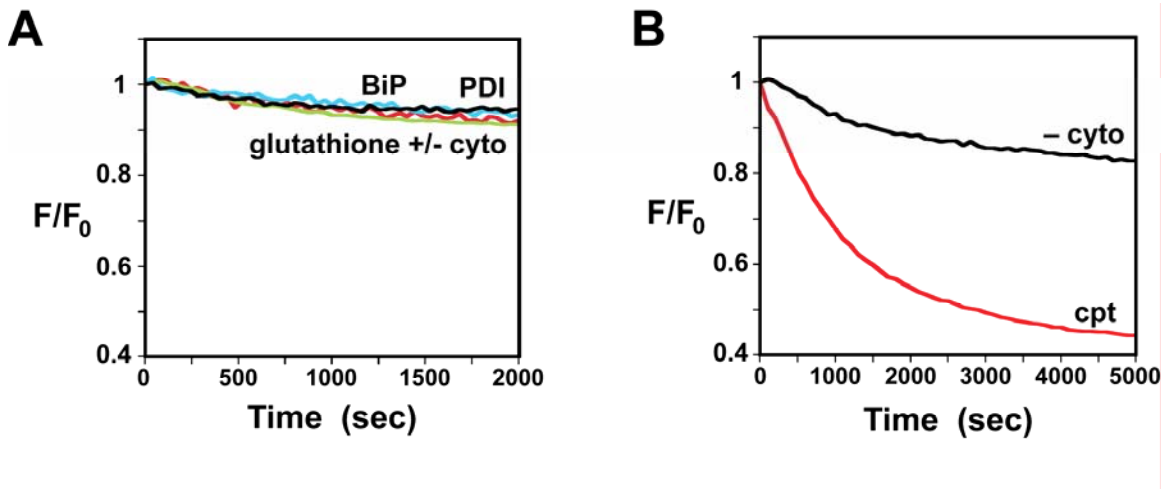

Figure S2. Retrotranslocation-Independent Exposure of BOF-Labeled Polypeptides to Cytosol

(A) RRMs containing ATP, total lumenal proteins, and either glutathione-BOF (red), PDI-BOF (black), or BiP-BOF (green) were incubated in cptcyto. RRMs containing glutathione-BOF were also incubated in the absence of cyto (cyan).

(B) CptRRMs were incubated for a longer time with cptcyto (red) or with no cytosolic proteins (black). For Figure S2, n = 3 to 5 independent experiments.

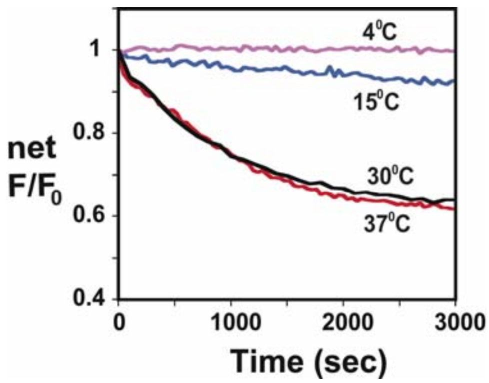

Figure S3. Temperature Dependence of Δgpαf-BOF Release

The average (n = 2) net αBOF-dependent quenching of Δgpαf-BOF due to retrotranslocation is shown at 4°C (magenta), 15°C (cyan), 30°C (black), or 37°C (red) after background subtraction [parallel samples of cptRRMS contained either cptcyto or 2 mM ATP (= background)].

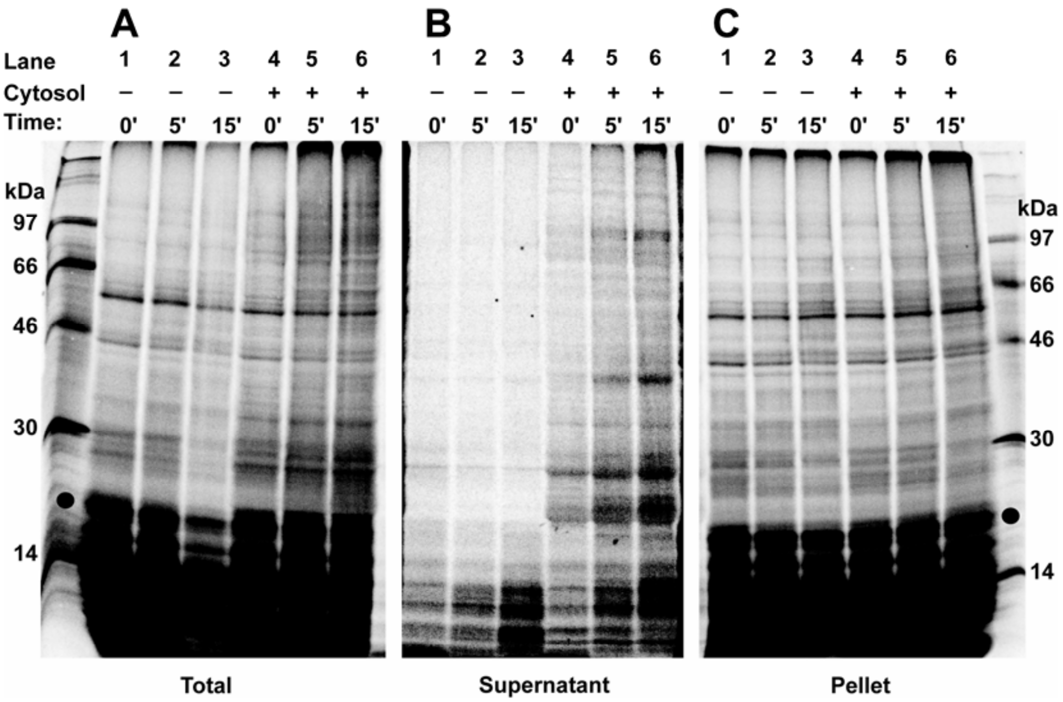

Figure S4. ΔgpαF Photocrosslinking to Lumenal, Cytosolic, and Membrane Components

pΔgpαf mRNA was translated in the presence of εANB-Lys-tRNA^{Lys} as in Figure 6 and translocated into microsomes in vitro in the dark. Microsomes were purified in the dark and then incubated in cptcyto at 30°C (0' samples were on ice throughout). Sample aliquots were photolyzed at the times indicated and then split in two. One half of each aliquot was analyzed directly by SDS-PAGE (A, total sample), while the other half was sedimented to separate the microsome pellet from the supernatant prior to SDS-PAGE analysis of the radioactive species in the supernatant (B) and the microsome pellet (C). Δgpαf, ●.
